# 17β-Estradiol activates Cl^−^ channels via the estrogen receptor α pathway in human thyroid cells

**DOI:** 10.1080/19336950.2021.1957627

**Published:** 2021-08-20

**Authors:** Meisheng Yu, Yuan Wei, Yanfang Zheng, Lili Yang, Long Meng, Jiawei Lin, Peisheng Xu, Sanaa Ahmed Nagi Abdu Mahdy, Linyan Zhu, Shuang Peng, Lixin Chen, Liwei Wang

**Affiliations:** a Department of Pathophysiology, Medical College, Jinan University, Guangzhou, China; b Center for Scientific Research and Institute of Exercise and Health, Guangzhou Sports University, Guangzhou, China; c Department of Physiology, Medical College, The Zhuhai Campus of the Zunyi Medical University, Zhuhai, China; d Academic Affairs Office, Guangzhou Medical University, Guangzhou, China; e Department of Obstetrics, Shiyan Maternal and Child Health Hospital, Hubei, Shiyan, China; f Department of Breast Surgery, The First People’s Hospital of Foshan, Foshan, China; g Department of Physiology, Medical College, Jinan University, Guangzhou, China

**Keywords:** Chloride channels, ClC-3, estrogen, estrogen receptor, thyroid

## Abstract

Estradiol regulates thyroid function, and chloride channels are involved in the regulation of thyroid function. However, little is known about the role of chloride channels in the regulation of thyroid functions by estrogen. In this study, the effects of estrogen on chloride channel activities in human thyroid Nthy-ori3-1 cells were therefore investigated using the whole cell patch-clamp technique. The results showed that the extracellular application of 17β-estradiol (E2) activated Cl^−^ currents, which reversed at a potential close to Cl^−^ equilibrium potential and showed remarkable outward rectification and an anion permeability of I^−^ > Br^−^ > Cl^−^ > gluconate. The Cl^−^ currents were inhibited by the chloride channel blockers, NPPB and tamoxifen. Quantitative Real-time PCR results demonstrated that ClC-3 expression was highest in ClC family member in Nthy-ori3-1 cells. The down-regulation of ClC-3 expression by ClC-3 siRNA inhibited E2-induced Cl^−^ current. The Cl^−^ current was blocked by the estrogen receptor antagonist, ICI 182780 (fulvestrant). Estrogen receptor alpha (ERα) and not estrogen receptor beta was the protein expressed in Nthy-ori3-1 cells, and the knockdown of ERα expression with ERα siRNA abolished E2-induced Cl^−^ currents. Estradiol can promote the accumulation of ClC-3 in cell membrane. ERα and ClC-3 proteins were partially co-localized in the cell membrane of Nthy-ori3-1 cells after estrogen exposure. The results suggest that estrogen activates chloride channels via ERα in normal human thyroid cells, and ClC-3 proteins play a pivotal role in the activation of E2-induced Cl^−^ current.

## Introduction

The thyroid gland is the largest endocrine gland in the human body. Its main function is to synthesize thyroid hormone, promote growth and development, and regulate metabolism [1]. Epidemiological studies on various thyroid diseases found a remarkable difference in the incidence rate of thyroid diseases between genders [2,3]. The prevalence of female hyperthyroidism is 0.5%–2%, which is 10 times that of male hyperthyroidism in the same area [4]. The incidence rate of thyroid cancer is 3–4 times higher in women than in men [5,6]. Animal studies showed that exogenous estrogen can promote the growth of thyroid tumor [7]. These studies suggest that estrogen may regulate thyroid hormone function and participates in the pathogenesis of thyroid diseases. Thyroid cells express estrogen receptors (ERα and ERβ) that are responsive to estrogen [4,8,9].

Chloride channels are the most important anion channels in the body. Chloride channels belong to a heterogeneous family of molecules, including the cystic fibrosis transmembrane conductance regulator (CFTR), calcium-activated chloride channel and voltage-gated chloride channel (ClC) and many more [10]. Increasing attention has been paid to the role of chloride channels in the thyroid gland. Studies have shown that CFTR may affect thyroid hormone levels [11,12]. ClC-3 is a member of the ClC family [13–15], which is regulated by many factors and participates in many physiological activities, such as cell volume regulation, cell cycle regulation, and apoptosis [16]. Estrogen has been shown to activate chloride channels in breast cancer cells and osteoblasts [17,18]. ClC-3 over expression in mice can lead to morphological changes in thyroid tissue and an increase in thyroid hormone secretion [19]. Thus, ClC-3 may be involved in the regulation of thyroid function.

Available evidence supports the notion that thyroid function is modulated by 17β-estradiol (E2) [20], and chloride channels are involved in the regulation of thyroid function [21,22]. However, the role of chloride channels in the estrogen regulation of thyroid function is poorly understood. In the present study, the effects of estrogen on chloride channel activities were investigated in human thyroid Nthy-ori3-1 cells.

## Methods

### Cell culture

Human normal transformed thyroid cell line (Nthy-ori3-1) cells were purchased from European Collection of Cell Cultures (agented by Guangzhou Jennio Biotech Co., Ltd.). Nthy-ori3-1 cells were routinely grown in RPMI 1640 medium (Gibco) supplemented with 10% fetal bovine serum (Gibco), 100 IU/ml penicillin, and 100 g/ml streptomycin (Sigma) in humidified atmosphere with 5% CO_2_ at 37°C. The cells were digested by trypsin and subcultured every 3 days.

### Solutions and chemicals

E2, tamoxifen, 5-nitro-2-(3′-phenylpropyl-amino) benzoic acid (NPPB), and ICI 182,780 (fulvestrant) were purchased from Sigma-Aldrich (USA), and all are dissolved in dimethyl sulfoxide as stock solutions (1, 20, 100 and 20 mmol/L, respectively), and diluted to final concentrations with isotonic bath solution. The final concentrations of E2, tamoxifen, NPPB, and ICI 182,780 were 1, 20, 100 and 100 μM, respectively. The pipette solution and isotonic bath solution were prepared as follows: for the pipette solution (in mM): 1.2 MgCl_2_, 1 EGTA, 10 HEPES, 70 N-methyl-D-glucamine chloride, 140 D-mannitol, and 2 ATP; for the isotonic bath solution (in mM): 70 NaCl, 2 CaCl_2_, 0.5 MgCl_2_, 10 HEPES, and 140 D-mannitol. The pH of the pipette and isotonic bath solutions were adjusted to 7.25 and 7.4 by Tris-base, respectively. For the anion substitution experiments, NaCl (70 mM) in the bath solution was substituted with an equimolar of NaBr, NaI, or sodium gluconate.

### Whole-cell current recording

The Nthy-ori3-1 cells were grown in culture flasks for 72 h until about 80% confluence. The cells were then harvested and resuspended, and plated onto glass coverslips and incubated for 1 h before the patch-clamp experiments. The coverslips with cells were placed in a perfused recording chamber. Changes in bath conditions were obtained by perfusing the cells with different bath solutions.

The whole-cell patch clamp technique was used to record the currents of Nthy-ori3-1 cells. The 22 mm round coverslip with Nthy-ori3-1 cells was placed in a bath chamber. Whole-cell currents were recorded with the EPC-7 patch-clamp amplifier (HEKA, Germany). Electrodes with a resistance of 5–10 M after filled with the pipette solution were made from glass capillaries (with an outer diameter of 1.5 mm) using a two-stage vertical puller (PB-7, Narishige, Japan). After the formation of whole cell configuration, the membrane potential was held at 0 mV (the chloride equilibrium potential) and stepped to ±40, 0, and ±80 mV for a 200 ms duration with 4 s interval between pulses. Command voltages and whole-cell currents were recorded simultaneously on a computer via a laboratory interface (CED 1401, Cambridge, UK) with a sampling rate of 3 kHz. All current measurements were made at 10 ms after the onset of each voltage step. The cells were continuously cycled through the voltage protocol in the experiments. Current densities were determined by normalizing the whole-cell current to the membrane capacitance. Current (*I*)–voltage (*V*) curves under different conditions and the curves of current–time function in a single experiment were plotted using the currents measured. The whole-cell currents of the cells with green fluorescence (indicates the successful transfection of 5′-carboxyfluorescein [FAM] fluorescein-labeled siRNA) were recorded with the patch-clamp technique under a florescent microscope (Olympus IX71). All experiments were carried out at room temperature (20–24°C).

In the anion substitution experiments, a “U”-shaped agar bridge was applied to minimize the baseline drift by connecting the reference electrode (Ag–AgCl wire) to the bath solution. When the E2-activated current reached the peak and leveled off, the bath solution containing 70 mM NaCl was substituted by an equimolar of NaI, NaBr,or sodium gluconate. Membrane current traces were recorded from the same cell for each anion substitution. The permeability ratios (*P*_X_/*P*_Cl_) of various anions (X^−^) relative to that of Cl^−^ were calculated using the modified Goldman–Hodgkin–Katz equation: *P*_X_/*P*_Cl_ = [(Cl^−^)_n_ exp(−Δ*E*_rev_ *F*/*RT*)−(Cl^−^)_s_]/(X^−^)_s_, where (Cl^−^)_n_ and [Cl^−^]_s_ are the Cl^−^ concentrations in the normal and substituted bath solutions, respectively; [X^−^]_s_ is the concentration of the substituted anion; Δ*E*_rev_ is the difference in the reversal potentials of Cl^−^ and X^−^; *F* is the Faraday constant (96480); *R* the gas constant (8.314); and *T* is the absolute temperature (273.16 + 20°C, room temperature). In these experiments, Cl^−^ concentration of the bath solution was decreased from 75 mM to 5 mM by replacing Cl^−^ with the substituted anion. E2 was added to the bath solution in the final concentration of 1 μM to activate Cl^−^ currents.

### Immunofluorescence analysis

Cells were grown on 6 mm round coverslips, then washed with PBS, and fixed with 4% paraformaldehyde for 15 min at room temperature. The cells were then rinsed with PBS and treated with 0.5% Triton X-100 for 5 min to permeabilize followed by blocking with 10% sheep serum for 45 min. Afterward, the cells were incubated with primary antibodies (rabbit anti-ClC-3 antibody, Cell Signaling Technology; mouse monoclonal anti-ClC-3 antibody, Abcam; rabbit anti-ERα antibody and rabbit anti-ERβ antibody, Abcam) at 4°C overnight. The cells were washed with 1% sheep serum and incubated with secondary antibody (Alexa Fluor 488-labeled goat anti-rabbit IgG, Cy3-labeled Goat Anti-Rabbit IgG (H + L), Alexa Fluor 488-labeled Goat Anti-Mouse IgG(H + L), Beyotime Institute of Biotechnology, China) for 1 h. The cells were rinsed with PBS and exposed to Hoechst 33,258 for 5 min to stain the nuclei. The cell side of the coverslips was attached to the mounting medium on glass slides. Immunofluorescence was detected under a Nikon C1-si confocal microscope (Japan).

### Quantitative real-time polymerase chain reaction (qRT-PCR)

The total RNA of the cultured Nthy-ori3-1 cells was extracted using RNAiso Plus reagent (Takara, Japan) and reverse transcribed to cDNA using the Prime Script RT Kit (Takara, Japan). cDNA was amplified using SYBR Premix Ex Taq^TM^ kit (TliRNaseH Plus; Takara, Japan) following the instructions of the manufacturer: initial step of 95°C/30 s, followed by 40 cycles of 95°C/5 s and 60°C/30 s, and melt analysis at 60–90°C at increments of 0.1°C/s. The primer sequences (Sangon Biotech, China) are as follows (Table 1). Housekeeping gene GAPDH was used as the reference.

**Table 1.** The primer sequences used for real-time PCR

Gene	Forward sequence (5ʹ to 3ʹ)	Reverse sequence (5ʹ to 3ʹ)	Product size(bp)
ClCN-1	GAATCCCCGAAATGAAGACA	TCCTACCAGCCTTCCAAATG	201
ClCN-2	GCTGTCATTGGTATTGCTAGTGG	AGCGTCTCTTTCTGTGAGAGCTGT	218
ClCN-3	TTGCCTACTATCACCACGAC	GCATCTCCAACCCATTTACT	226
ClCN-4	CCCTGGTACATGGCTGAACT	CTCTGGCGTGTGTAGGGATT	203
ClCN-5	TGGACTCCTCCAAGCTCTGT	AGGCCAGAAGGGATCTTCAT	178
ClCN-6	ATTTGGGTTTCTTCGTCGTG	CGGCATTCTCCTAACACCAT	202
ClCN-7	GGAGAAAATGGCCTACACGA	AGATCAGCACGAAGGCAACT	203
GADPH	GGTGGTCTCCTCTGACTTCAACA	GTTGCTGTAGCCAAATTCGTTGT	127

### SiRNA technique

SiRNAs were synthesized by Gene Pharma (China). SiRNAs labeled with FAM were used in whole-cell recording. The sense and antisense strands of ClC-3 siRNA are 5′-CAA UGG AUU UCC UGU CAU ATT-3′ and 5′-UAU GAC AGG AAA UCC AUU GTA-3′, respectively. The sense and antisense strands of ERαsiRNA are 5′-GGU CCA CCU UCU AGA AUG UTT-3′ and 5′-ACA UUC UAG AAG GUG GAC CTT-3′, respectively. The sense and antisense strands of negative control siRNA were 5′-UUC UCC GAA CGU GUC ACG UTT-3′ and 5′-ACG UGA CAC GUU CGG AGA ATT-3′, respectively. The cells at 50%–60% confluency were transfected with 100 nM ClC-3 siRNA, ER siRNA, or negative control siRNA plus siRNA-Mate^TM^ at 1/250 final dilution (Gene Pharma, China) and then cultured in normal medium for 48 h.

### Western blot

Whole cell lysates were prepared with radio immunoprecipitation assay lysis buffer. Total proteins were isolated and quantified by bicinchoninic acid protein assay (KeyGen Biotech, China). The proteins were boiled in the loading buffer, electrophoresed on 10% sodium dodecyl sulfate–polyacrylamide gel, transferred onto nitrocellulose membranes, and blocked in 5% nonfat dry milk. Afterward, the cells were incubated with primary antibody (mouse anti-ClC-3, Abcam; rabbit anti-ERα, Abcam) and then with horseradish peroxidase-labeled secondary antibody (goat anti-mouse IgG, goat anti-rabbit IgG; Invitrogen). The target proteins were detected by chemiluminescence detection method.

### Statistical analysis

Statistical analysis was performed using SPSS 17.0 software (SPSS, USA). The experimental data are expressed as mean ± standard error (SE). Student’s t-test was employed to analyze the significance of differences in the levels of Cl^−^ currents between groups. One-way ANOVA was used to test the significance of differences in the levels of mRNA and protein expression between groups. *P* < 0.05 was considered statistically significant.

## Results

### E2 activated a chloride current in Nthy-ori3-1 cells

**Figure 1.**
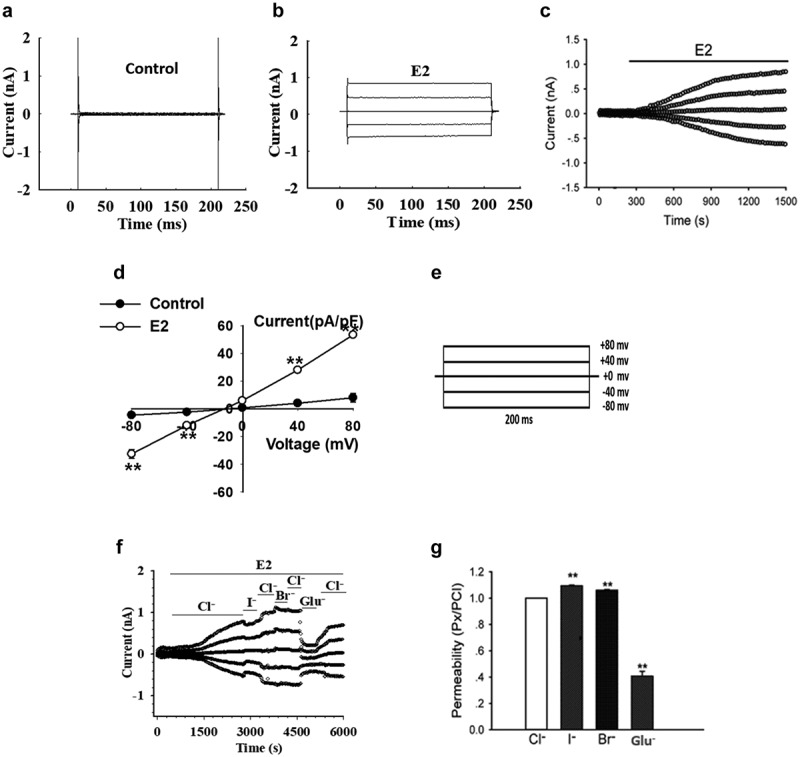
17β-estradiol (E2) activated Cl^−^ currents in human thyroid Nthy-ori3-1 cells. (a) and (b) The typical current traces recorded under the isotonic bath solution (Control) and after extracellular application of 1 µM 17β-estradiol (E2), respectively. (c)The typical time course of the E2-activated current. (d) The current–voltage (i–v) relationships under different treatment conditions (n = 5, **P < 0.01, vs Control). (e) Voltage protocol used in whole–cell patch clamp recording. The voltage is hold at 0 mV and then stepped to 0, ± 40, ± 80 mV with an interval of 4 sec between steps. (f) The typical time course of E2-induced currents for different permeable anions. (g) The permeability ratio of I^−^, Br^−^, and gluconate^−^ to Cl^−^ for the E2-activated chloride channels (mean ± SE, n = 5, **P < 0.01 vs Cl^−^)

The E2-induced Cl^−^ current in Nthy-ori3-1 cells were recorded using the whole cell patch-clamp technique. As shown in Figure 1a, the background currents in response to all voltage pulses were small and stable when the cell was bathed in control solution (E2-free solution). The application of E2 (1 µM) to the bath solution activated a current in 5–10 min. The E2-activated current showed an obvious outward rectification property but had no apparent time- or voltage-dependent inactivation when the potentials were imposed (Figure 1b). The time course of the E2-activated whole-cell current is shown in Figure 1c. The current reached a peak in 10–20 min with the density of 53.4 ± 1.7 pA/pF at +80 mV and −32.6 ± 3.2 pA/pF at −80 mV (n = 5, *P* < 0.01, vs control solution, Figure 1d). The *I–V* relationship demonstrated that the E2-activated current is reversed at a voltage close to the calculated Cl^−^ equilibrium potential (−0.9 mV) with a mean value of −4.0 ± 0.3 mV (n = 5, Figure 1d). The protocol in voltage clamp is shown in Figure 1e. The voltage was held at 0 mV and then stepped to 0, ±40, and ±80 mV with an interval of 4 s between steps. K^+^ was eliminated from the pipette and bath solutions in the experiments, thus, the data strongly support the hypothesis that the E2-activated current was carried out by chloride.

The anion permeability of the E2-induced current was tested by replacing external NaCl with Na(X), where X represents the substitution anion: I^−^, Br^−^, or gluconate (Glu^−^).When the E2-induced currents were activated and reached their peaks, the bath solution with 70 mM Cl^−^ was replaced by the solution containing an equimolar of I^−^, Br^−^, or gluconate. Membrane current traces in response to voltages from −80 mV to 80 mV were recorded from the same cell for each anion substitution (figure 1f).The modified Goldman–Hodgkin–Katz equation was used to calculate the shifts in reversal potential. The permeability ratios, P_I_/P_Cl_, P_Br_/P_Cl_, and P_gluconate_/P_Cl_, were 1.09 ± 0.01, 1.06 ± 0.01, and 0.41 ± 0.04 (n = 5) respectively, which show that the sequence of anion permeability is: I^−^ > Br^−^ >Cl^−^ >gluconate (Figure 1g).

### Pharmacological properties of E2-induced chloride currents in Nthy-ori3-1 cells

**Figure 2.**
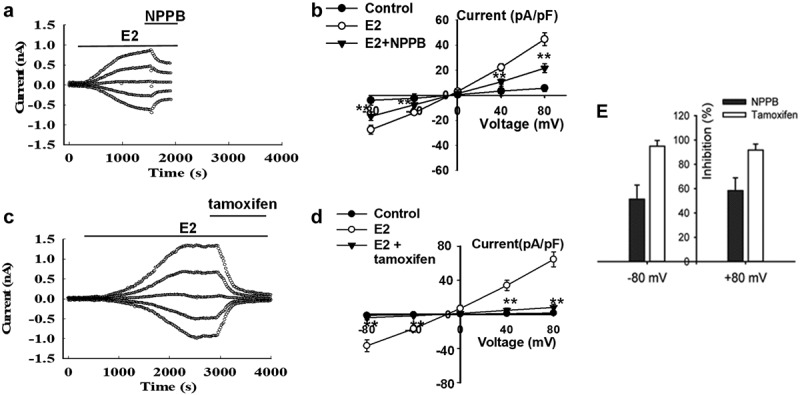
The chloride channel blockers inhibited the currents induced by E2. (a) and (c) Inhibitory effects of the chloride channel blockers, NPPB (100 µM) and tamoxifen (20 µM), on the E2-activated currents, respectively. (b) and (d) The I–V relationships under different treatment conditions (mean**±** SE, n = 6 to 14, **P < 0.01 *vs* E2). (e) Inhibition rate of NPPB and tamoxifen on E2-activated current

The effects of the chloride channels blockers, NPPB and tamoxifen, on the E2-activated current were observed to further unravel the pharmacological properties of the E2-induced current. The cells were exposed to E2 solution to induce Cl^−^ currents when the current induced by 1 µM E2 reached the peak and was maintained at a relatively stable plateau, the solution that contained the indicated blockers was applied. Figure 2a presents the time course of the effects of NPPB on the currents. The extracellular application of 100 µM NPPB remarkably suppressed the E2-induced current. The *I*–*V* relationships are shown in Figure 2b. Outward and inward currents were blocked with inhibition rates of 58.4% ± 10.6% at +80 mV and 51.3% ± 11.6% at −80 mV (n = 14, *P* < 0.01). Tamoxifen also inhibited the outward and inward components of the E2-induced currents (Figure 2c,e). Compared with NPPB, tamoxifen showed a stronger inhibitory effect on the current with inhibitory rates of 94.9% ± 4.7% at −80 mV and 91.7% ± 5.0% at +80 mV (n = 6, *P* < 0.01, Figure 2d,e).

### Knockdown of ClC-3 expression suppressed E2-induced Cl^−^ currents

**Figure 3.**
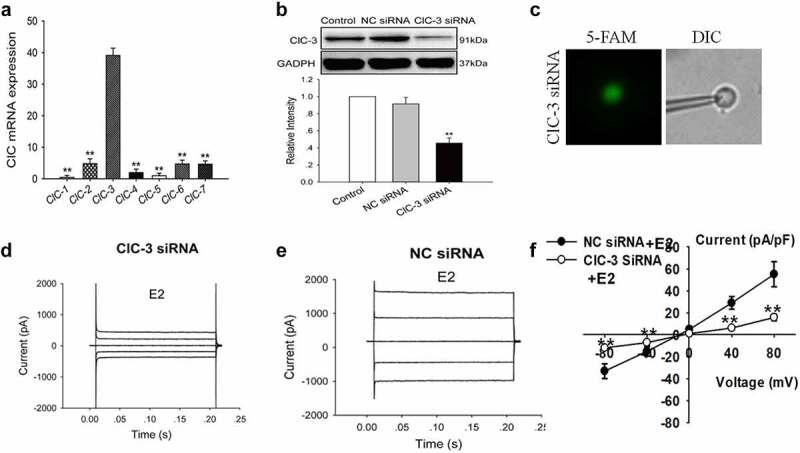
ClC-3 protein expression and E2-activated chloride currents were decreased by ClC-3 siRNA in Nthy-ori3-1 cells. (a) Quantitative analysis of ClC mRNA expression (mean ± SE, *n* = 3, **P < 0.01, *vs* ClC-3). (b) Knockdown ClC-3 proteins expression by ClC-3siRNA (ClC-3siRNA), but not by the negative control siRNA (NC-siRNA) (n = 3, **P < 0.01, *vs* Control). The Cl^−^ currents of the cells with green fluorescence (indicating successful transfection of the FAM carboxy fluorescein-labeled siRNA) were recorded with the patch clamp technique under the fluorescence microscope. (c) Recording pipettes and the siRNA-transfected cells with green fluorescence. (d) and (e) The typical current traces of E2-induced Cl^−^ currents in the cells treated with the ClC-3 siRNA and NC-siRNA, respectively. (f) I–V relationships of the E2-induced currents in ClC-3 siRNA and NC-siRNA groups (mean± SE, n = 5, **P < 0.01 *vs* NC-siRNA)

The ClC family has nine members in mammals. The ClC family has differential tissue distribution and function. However, the expression level of the different isoforms of the ClC family in the thyroid is still unclear. In this study, the mRNA expression of ClC family members in Nthy-ori3-1 cells was investigated by qRT-PCR. The results showed the mRNA expression levels of *ClC1–7* genes in Nthy-ori3-1 cells, and the expression of *ClC-3* was the highest (n = 3, *P* < 0.01, Figure 3a).

The role of ClC-3 in the activation of E2-activated Cl^−^ current was further investigated. The expression of ClC-3 proteins in Nthy-ori3-1 cells was knocked down by siRNA. As shown in Figure 3b, the expression of ClC-3 proteins detected by Western blot was remarkably reduced after treatment with ClC-3 siRNA for 48 h, whereas treatment with negative control siRNA (NC siRNA) did not show a substantial effect on ClC-3 expression (Figure 3b).

The chloride currents of the cells with green fluorescence (which indicates the successful transfection of FAM-labeled siRNA) were recorded using the patch-clamp technique under a fluorescence microscope (Figure 3c). The results indicated that the downregulation of ClC-3 expression attenuated the activation of E2-activated Cl^−^ current. The E2-activated Cl^−^ current in the ClC-3-siRNA group was 15.8 ± 3.0 pA/pF at 80 mV, which was significantly lower than that in the NC siRNA group (55.1 ± 11.4 pA/pF, n = 5, *P* < 0.01) (Figure 3d-f). The results suggest that ClC-3 may be the main chloride channels activated by E2 in Nthy-ori3-1 cells.

### Estrogen receptors were involved in the activation of Cl^−^ currents induced by E2

Estrogen binds to ER and induces the activation of estrogen signal transduction pathway. ICI 182780 (fulvestran) is an effective ER antagonist, which competitively inhibits the binding of estradiol to ER. We examined the effect of ICI 182780 on E2-induced Cl^−^ current to investigate whether estrogen activates the Cl^−^ channels through ER.

**Figure 4.**
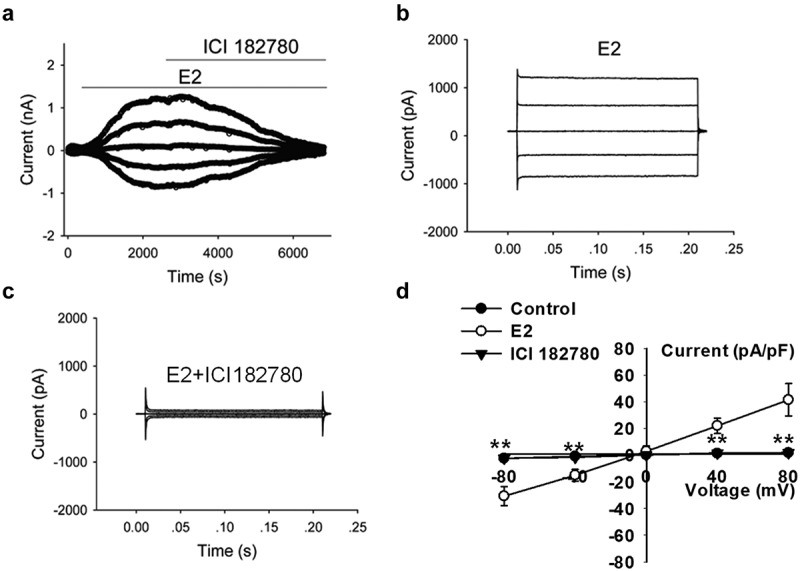
Estrogen receptor antagonist ICI 182,780 suppressed the E2-induced Cl^−^ currents. (a) The typical time course of E2-induced current and inhibitory effects of the extracellular application of 100 µM ICI 182,780 on the currents. Typical current traces of (b) E2-induced Cl^−^ currents and (c) E2-induced Cl^−^currents in the cells treated with ICI 182,780. (d) The I–V relationships of the E2-activated currents under different treatment conditions (mean± SE, n = 4, **P < 0.01, *vs* E2)

The results showed that the extracellular application of ICI 182,780 remarkably suppressed the chloride currents induced by E2 challenge at all the voltage steps applied (0, ±40, and ±80 mV). Figure 4a presents the time course of the effect of ICI 182780 on E2-induced current. When the chloride current activated by E2 reached a stable peak, 100 μM ICI 182780 dissolved in the bath solution containing E2 was perfused through the bath. Figure 4b,c show the typical traces of E2-induced whole-cell currents and the inhibitory effects of 100 μM extracellular ICI 182780 on the currents.

The *I–V* relationships under control (without E2) and E2 conditions, as well as E2 plus ICI 182780 treatment, are shown in Figure 4d. The inward and outward currents activated by E2 were suppressed by ICI 182780. ICI 182780 inhibited 95.6% ± 3.8% of the inward current induced by −80 mV and 97.4% ± 1.9% of the outward current induced by +80 mV (n = 4, *P* < 0.01 *vs* E2). No remarkable difference in the inhibition of inward and outward currents was noted. The results suggest that E2 activates chloride channels through the estrogen receptors.

### E2 activated Cl^−^ channels via the ERα pathway

**Figure 5.**
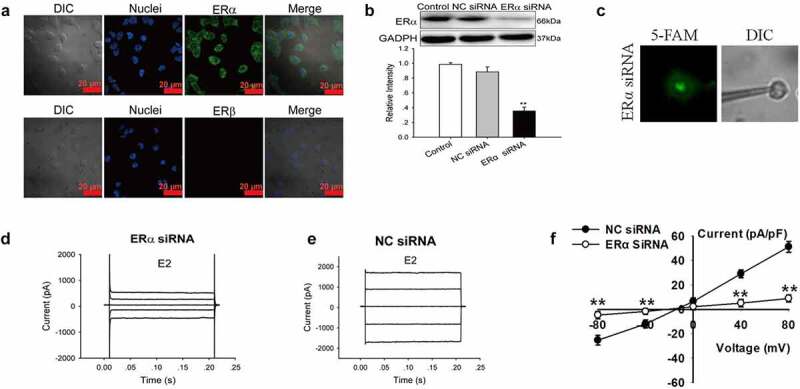
Knockdown of ERα expression abolished the E2-induced Cl^−^ currents. (a)ERα and ERβ expression in Nthy-ori3-1 cells detected by immunofluorescence. ERα was present in Nthy-ori3-1 cells, where as ERβ was absent. (b) ERα protein expression examined by Western blot. Bar charts show the down-regulation of ERα protein expression by ERα siRNA but not by NC siRNA (mean ± SE, *n* = 3, **P < 0.01, *vs* Control). (c) Recording pipettes and the siRNA-transfected cells with green fluorescence. (d) and (e) The typical current traces of the E2-induced Cl^−^ currents in the cells treated with ERα siRNA and NC siRNA, respectively. (e) The I–V relationships of the E2-induced currents in ERα siRNA and NC siRNA groups (mean± SE, *n* = 5, **P < 0.01, *vs* NC siRNA)

Estrogen works by binding to ERα or ERβ. We investigated the expression of ERα and ERβ in Nthy-ori 3–1 cells by immunofluorescence to determine whether ER plays a role in the activation of chloride channels. As shown in Figure 5a, ERα was present in Nthy-ori3-1 cells, whereas ERβ was absent. We further investigated the role of ERα in E2-induced chloride current by siRNA technology. The expression of ERα protein decreased significantly after treatment with ERα siRNA for 48 h (three experiments, *P* < 0.01, *vs* NC siRNA, Figure 5b).

The Cl^−^ currents in the cells successfully transfected with 5′-FAM-conjugated ERαsiRNA (labeled with green fluorescence) were recorded by patch-clamp technique under a fluorescence microscope (Figure 5c). The results showed that the E2-induced Cl^−^ currents were attenuated substantially by ERα siRNA. As shown in Figure 5d and E, E2 activated a small current in the cells successfully transfected with ERα siRNA. In the ERα siRNA group, E2-activated Cl^−^ current was 8.9 ± 3.9 pA/pF (+80 mV), which was significantly lower than those in NC siRNA group (51.2 ± 4.5 pA/pF, +80 mV, n = 5, *P* < 0.01) (figure 5f). The results indicated that ERα was involved in the activation of Cl^−^ current induced by E2.

### Estrogen promoted the aggregation of ClC-3 in the cell membrane and the colocalization of ERα and ClC-3

**Figure 6.**
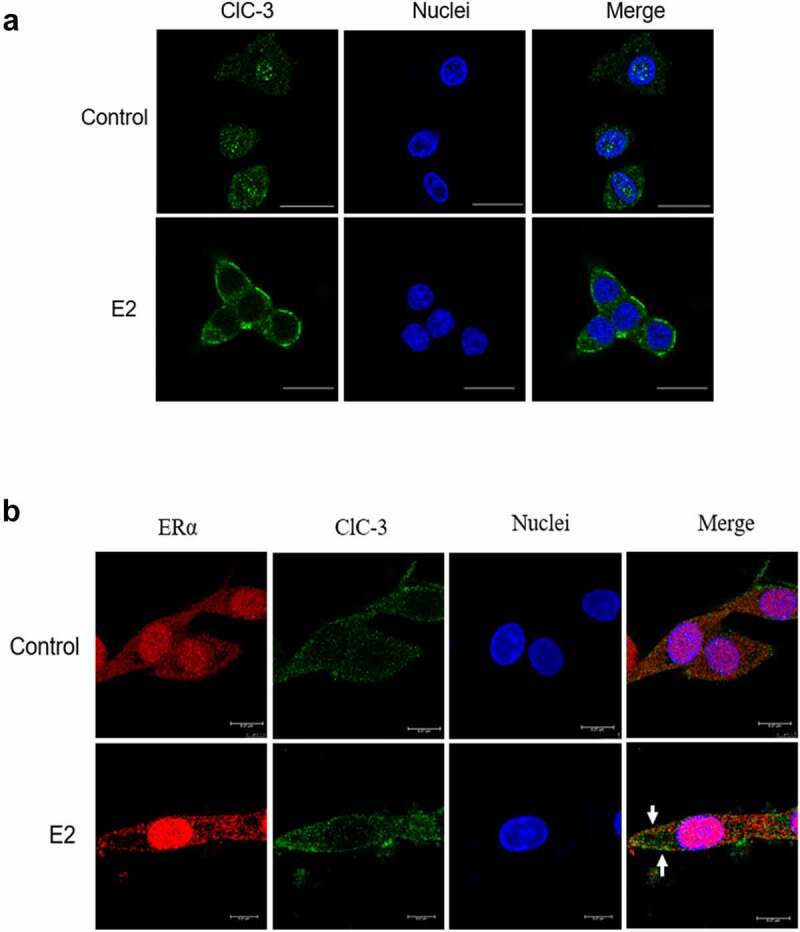
Estrogen promotes the accumulation of ClC-3 in the cell membrane and co- localization of ERα and ClC-3. The localization of ClC-3 (labeled with AlexaFluor 488, green) and ERα (labeled with Cy-3, red) were detected by immunofluorescence under the confocal microscope. (a) The ClC-3 fluorescence (green) of the cells in the control cells and the E2-treated cells. The white bar corresponds to 20 μm. (b) Co-localization of ER α (red) and ClC-3 (green) in E2-treated cells. The white bar corresponds to 7.5 μm. The arrow points colocalization of ClC-3 and ERα

The results suggest that ClC-3 may be the main chloride channels activated by E2 in Nthy-ori3-1 cells. ClC-3 is predominantly located inside cells, and a small amount exists in the cell membrane. We determined whether estradiol induces the trafficking of channel protein to the plasma membrane and whether ER interacts with ClC-3 in the cell membrane. The localization of ClC-3 and the colocalization of ERα and ClC-3 were detected by immunofluorescence and immunofluorescence colocalization analysis respectively. As shown in Figure 6a (Control), ClC-3 proteins (labeled with Alex Fluor 488, green) were distributed inside the cell. However, in the cells treated with 1 μM E2 for 10 min, the fluorescence of ClC-3 was remarkably enhanced in the cells membrane as presented in Figure 6a (E2).

As presented in Figure 6b, ERα (labeled with Cy-3, red) and ClC-3 (labeled with Alex Fluor 488, green) were distributed in the nucleus and cytoplasm of Control cells. The expression of ClC-3 and ERα increased in the cell membrane of the cells treated with 1 μM E2 for 10 min compared with the control. The partly overlaps of ClC-3 and ERα appeared in the cell membrane. The results indicated that estrogen can induce the translocation and colocalization of ERα and ClC-3 to the cell membrane.

## Discussion

The incidence rate of thyroid diseases in women is remarkably higher than that in men [23]. The difference in the incidence rates between gender suggests that thyroid diseases may be affected by sex hormones, especially estrogen. However, the exact pathogenesis is still unclear. Current studies found that estrogen can play a role by affecting the opening or closing of the ion channels [24]. However, the effect of estrogen on the chloride channels of thyroid cells is rarely studied.

In this present study, we demonstrated the E2 activated Cl^−^ currents in human thyroid Nthy-ori3-1 cells. We found that the background currents were small and stable when the cell was bathed in control solution. The extracellular application of 1 µM E2 activated a large chloride current which the current possessed obvious outward rectification characteristics. The ionic selectivity of the current was in the order: I^−^ > Br^−^ > Cl^−^ > gluconate. The results showed that the channel activated by E2 has high permeability to Cl^−^, I^−^, and Br^−^. The reversal potential of the E2-induced current was close to the calculated Cl^−^ equilibrium potential. These data suggested that the E2-induced current was a Cl^−^ current. The result, as well as the determined inhibitory effects of the chloride channels blockers, tamoxifen and NPPB, on the current supported that chloride channels were the target of E2.

ClC-3 is considered a Cl^−^/H^+^ exchanger in intracellular vesicles and a chloride channels in the cell membrane [15,25]. Our previous study demonstrated that ClC-3 contributes to the Cl^−^ currents induced by extracellular acidification or estrogen [18,26]. ClC-3 may be one of the action targets of estrogen in the regulation of osteoblastic activities [18]. In the present study, the results showed that ClC-3 had the highest expression level among the ClC family in Nthy-ori3-1 cells. The downregulation of ClC-3 expression by ClC-3 siRNA suppressed the E2-induced Cl^−^ current; thus, ClC-3 protein acts as the main channel protein in the activation of E2-induced Cl^−^ current in Nthy-ori3-1 cells.

ClC-3 was predominantly distributed in the nucleus and cytoplasm [27]. However, the distribution of ClC-3 can be regulated. The location of ClC-3 could be changed under different physiological or pharmacological conditions. The ClC-3 in the plasma membrane can rapidly endocytose into an intracellular site [13]. In addition, when cells were treated with 17β-E2 for 30 min, ClC-3 protein could translocate from the nucleus to cell membrane [17]. The subcellular distribution of endogenous ClC-3 varies in a cell cycle-dependent manner in HeLa cells [27]. Estrogen promoted the translocation of ClC-3 to the cell membrane in osteoblastic MC3T3-E1 cells [18].

Our results showed that the background current is small and stable in isotonic bath solution (without E2). However, the application of E2 activated a large chloride current. The whole-cell current is formed by the opening of ion channels on the cell membrane and the flow of ions across the membrane; therefore, we determined whether estradiol can increase the distribution of ClC-3 channels in the activation of the current. The results indicated that the fluorescence intensity of ClC-3 protein in the cell membrane was remarkably enhanced after treatment with estradiol. This finding suggested that estradiol can promote the accumulation and opening of ClC-3 channels on the cell membrane and produce large chloride currents in Nthy-ori3-1 cells.

Estrogen acts on different ion channel types and tissues through different signaling pathways with or without reliance on ERα expression [24]. We verified that ClC-3 protein is the main component of E2-activated Cl^−^ channels in ERα-positive breast cancer MCF-7 cells [17] and osteoblastic MC3T3-E1 cells [18]. In the present study, we found that the ER blocker, ICI 182780, almost completely inhibited the E2-induced current. This result indicates that E2 activates chloride channels through ERs. We further investigated the subtype of ERs. The results showed that ERα but not ERβ was the main protein expressed in Nthy-ori3-1 cells. The downregulation of ERα expression by siRNA abolished the E2-induced Cl^−^ currents. These data indicated that E2 activates chloride channels via the ERα-dependent pathway.

Classical ER, including ERα and ERβ, are distributed in the nucleus. However, increasing evidence suggest that classical ER can also be seen in the cell membrane [17]. ERα in MCF-7 cells is translocated to the cytoplasm and cell membrane when exposed to E2 [17]. Similar results were observed in osteoblastic MC3T3-E1 cells [18]. ERα accumulates in the cell membrane after treatment with estrogen in MC3T3-E1 cells [18].These finding imply that ER is translocated to the cell membrane when exposed to estrogen and forms an E2–ERα complex, which activates the chloride channels in the cell membrane. We further studied the interaction of ERα and ClC-3. The result showed that the two proteins were colocalized in the cell membrane of Nthy-ori3-1 cells exposed to estrogen. However, more future work should be done to clarify their relationships.

In conclusion, estrogen activates chloride channels via ERα in human thyroid Nthy-ori3-1 cells. ClC-3 proteins play a pivotal role in the activation of estrogen-induced chloride current.
